# Feasibility of the deep learning method for estimating the ventilatory threshold with electrocardiography data

**DOI:** 10.1038/s41746-020-00348-6

**Published:** 2020-10-29

**Authors:** Kotaro Miura, Shinichi Goto, Yoshinori Katsumata, Hidehiko Ikura, Yasuyuki Shiraishi, Kazuki Sato, Keiichi Fukuda

**Affiliations:** 1 Department of Cardiology, Keio University School of Medicine, Tokyo, Japan; 2 Institute for Integrated Sports Medicine, Keio University School of Medicine, Tokyo, Japan

**Keywords:** Cardiovascular diseases, Rehabilitation

## Abstract

Regular aerobic physical activity is of utmost importance in maintaining a good health status and preventing cardiovascular diseases (CVDs). Although cardiopulmonary exercise testing (CPX) is an essential examination for noninvasive estimation of ventilatory threshold (VT), defined as the clinically equivalent to aerobic exercise, its evaluation requires an expensive respiratory gas analyzer and expertize. To address these inconveniences, this study investigated the feasibility of a deep learning (DL) algorithm with single-lead electrocardiography (ECG) for estimating the aerobic exercise threshold. Two hundred sixty consecutive patients with CVDs who underwent CPX were analyzed. Single-lead ECG data were stored as time-series voltage data with a sampling rate of 1000 Hz. The data of preprocessed ECG and time point at VT calculated by respiratory gas analyzer were used to train a neural network. The trained model was applied on an independent test cohort, and the DL threshold (DLT; a time of VT estimated through the DL algorithm) was calculated. We compared the correlation between oxygen uptake of the VT (VT–VO_2_) and the DLT (DLT–VO_2_). Our DL model showed that the DLT–VO_2_ was confirmed to be significantly correlated with the VT–VO_2_ (*r* = 0.875; *P* < 0.001), and the mean difference was nonsignificant (−0.05 ml/kg/min, *P* > 0.05), which displayed strong agreements between the VT and the DLT. The DL algorithm using single-lead ECG data enabled accurate estimation of VT in patients with CVDs. The DL algorithm may be a novel way for estimating aerobic exercise threshold.

## Introduction

Adequate regular physical activity is paramount to maintaining good health^1,2^ and preventing cardiovascular diseases (CVDs)^3,4^. In contrast, an unexpected high-intensity exercise could be a cause of deterioration or hospitalization in patients with CVDs^5,6^; current clinical practice guidelines and expert statements recommend aerobic exercise for patients with CVDs^7,8^. Although cardiopulmonary exercise testing (CPX) is an essential examination for noninvasively detecting the ventilatory threshold (VT), that is, defined as clinically equivalent to aerobic exercise, its assessment requires an expensive respiratory gas analyzer and expertize. In this context, expansion of the exercise therapy with a simple, versatile methodology to facilitate its introduction and persistency is warranted to improve clinical outcomes of patients with CVDs.

Advancement of high-performance computer technology and deep learning (DL) technology has enabled generation of models that accurately predict outcomes, detect diseases, and automatically classify or quantitate measurements from various modalities including electrophysiological and imaging data^9^ (e.g., electrocardiography [ECG]^10^, echocardiography^11^, computed tomography^12^, single-photon emission computed tomography^13^, and magnetic resonance imaging^14^) in cardiovascular medicine. In addition, DL aids in the interpretation of clinically important findings from imaging data to support clinical judgment by physicians^15^. However, there is limited evidence for estimating the threshold of aerobic exercise with DL algorithms combined with neural networks. Herein, we aimed to investigate the feasibility of a DL algorithm with single-lead ECG during incremental exercise for estimating the aerobic exercise threshold in patients with CVDs.

## Results

### Patients’ selection

**Fig. 1:**
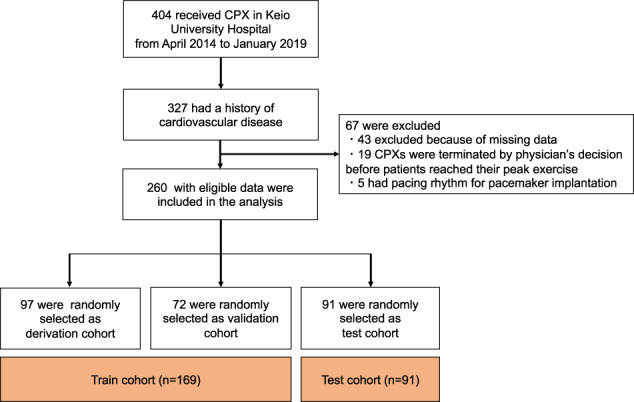
Patient flow chart. CPX cardiopulmonary exercise testing.

From April 2014 to May 2019, 404 patients underwent CPX in Keio University Hospital (Fig. 1). Among patients who were eligible for screening, we extracted 327 patients who had CVDs (chronic heart failure, coronary artery disease, pulmonary hypertension, or arrhythmias). The exclusion criteria of this study were patients with missing data, patients whose CPXs were terminated at the physician’s discretion before peak exercise load, and patients with pacing rhythm due to implantation of a pacemaker or a cardiac resynchronization therapy device. Two hundred sixty individuals who had CVD with eligible data were included in the final analysis.

### Patient characteristics

Tables 1 and 2 show the baseline characteristics and respiratory gas data during CPX of the train and test cohorts. Overall, the patients were predominantly men (73.1%), with an average age of 58.9 ± 14.6 years, and mean body mass index was 23.5 ± 3.9 kg/m^2^. Comorbidities included hypertension (46.5%), diabetes mellitus (18.1%), and dyslipidemia (40.4%). Medical history of CVD included chronic heart failure (46.5%), coronary artery disease (38.4%), pulmonary hypertension (15.8%), and arrhythmias (14.2%). The details of each medical history are provided in Supplementary Table 1. Twenty-five (9.6%) patients had atrial fibrillation (AF) during CPX. There were no significant differences in patients’ backgrounds among the two cohorts (the train cohort and the test cohort). In respiratory gas analysis data, there was significant difference of the oxygen uptake at VT (VT–VO_2_) in the train and test cohort (14 ± 4.5 vs. 15.7 ± 5.8 mL/kg/min; *P* = 0.041, Table 2).

**Table 1:** Baseline characteristics stratified by the train or test cohort.

	All patients (*n* = 260)	Train cohort (*n* = 169)	Test cohort (*n* = 91)	*P* value^*^
Age, years	58.9 ± 14.6	58.6 ± 14.5	59.4 ± 14.9	0.720
Male sex	190 (73.1%)	122 (72.2%)	68 (74.7%)	0.660
Height, cm	165.6 ± 8.6	165.4 ± 8.5	165.8 ± 8.7	0.552
Weight, kg	64.7 ± 13.8	64.2 ± 14.0	65.7 ± 12.8	0.289
BMI, kg/m^2^	23.5 ± 3.9	23.3 ± 3.9	23.8 ± 3.8	0.272
Hypertension	121 (46.5%)	78 (46.2%)	43 (47.3%)	0.865
Diabetes mellitus	47 (18.1%)	33 (19.5%)	14 (15.4%)	0.532
Dyslipidemia	105 (40.4%)	75 (44.4%)	30 (33.3%)	0.074
Chronic heart failure	121 (46.5%)	78 (46.2%)	43 (47.3%)	0.865
Coronary artery disease	100 (38.4%)	70 (41.4%)	30 (33.0%)	0.181
Pulmonary hypertension	41 (15.8%)	22 (13.0%)	19 (20.9%)	0.097
Arrhythmias	37 (14.2%)	14 (15.4%)	23 (13.6%)	0.696
Prescription of a β-blocker	177 (68.1%)	122 (72.2%)	55 (60.4%)	0.142
AF rhythm during CPX	25 (9.6%)	17 (8.8%)	8 (10.1%)	0.741

Values are presented as a mean ± standard deviation or number (percentage).

^*^Difference between the train cohort and the test cohort for each item.

*BMI* body mass index, *AF* atrial fibrillation, *CPX* cardiopulmonary exercise testing.

**Table 2:** Cardiopulmonary exercise testing data of patients in the train and test cohorts.

	Rest	VT	Peak
*Train cohort (n* *=* *169)*			
HR, bpm	75 ± 13	103 ± 18	136 ± 27
SBP, mmHg	118 ± 18^*^	146 ± 26	167 ± 36^*^
DBP, mmHg	74 ± 13	80 ± 12	83 ± 17
VO_2_, mL/kg/min	3.9 ± 0.8	14.0 ± 4.5^*^	21.8 ± 7.4
WR, W	–	56 ± 26	106 ± 45
RQ	–	0.97 ± 0.70	1.17 ± 0.11
VE-VCO_2_ slope	30.2 ± 5.8	–	–
*Test cohort (n* *=* *91)*			
HR, bpm	76 ± 13	107 ± 18	138 ± 27
SBP, mmHg	123 ± 20^*^	151 ± 29	173 ± 41^*^
DBP, mmHg	77 ± 13	83 ± 13	83 ± 16
VO_2_, mL/kg/min	4.0 ± 0.8	15.7 ± 5.8^*^	23.1 ± 8.0
WR, W	–	62 ± 28	114 ± 47
RQ	–	0.91 ± 0.12	1.16 ± 0.12
VE–VCO_2_ slope	29.7 ± 5.3	–	–

Values are presented as a mean ± standard deviation.

^*^P < 0.05, difference between the train cohort and the test cohort for each item.

*VT* ventilatory threshold, *HR* heart rate, *SBP* systolic blood pressure, *DBP* diastolic blood pressure, *VO*_*2*_ oxygen uptake, *WR* work rate, *RQ* respiratory quotient, *VE–VO*_*2*_ slope ventilation–carbon dioxide production slope, *bpm* beats per minute.

### Evaluation of the DL algorithm to estimate the threshold of aerobic exercise

**Fig. 2:**
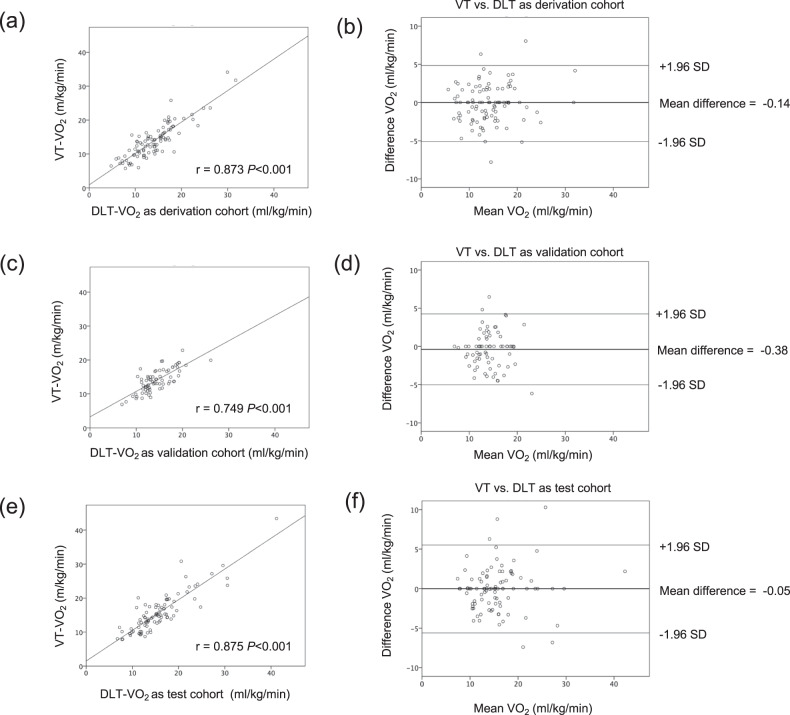
Validity testing of the VO_2_ at the ventilatory threshold of the derivation, validation, and test cohorts in patients with cardiovascular diseases. The graphs in the left panel show the relationship between the VT–VO_2_ and the DLT–VO_2_ for each cohort (**a**, **c**, **e**). The graphs in the right panel show the Bland–Altman plots (**b**, **d**, **f**), which indicate the respective differences between the VT–VO_2_ and at the DLT–VO_2_ for each cohort (*y*-axis) against the mean of the VT–VO_2_ and at the DLT–VO_2_ for each cohort (*x*-axis). The thinner horizontal lines in each Bland–Altman plot represent a ±1.96 SD. VO_2_ oxygen uptake, DLT deep learning threshold, VT ventilatory threshold, SD standard deviation.

The VO_2_ estimated from a single-lead ECG using DL algorithm (DL threshold, DLT–VO_2_) was compared with the VT–VO_2_ manually detected from a respiratory gas analyzer during CPX. The relationship between the VT–VO_2_ and the DLT–VO_2_ of the train cohorts showed a satisfactory result (derivation cohort: *r* = 0.873, *P* < 0.001, and validation cohort: *r* = 0.749, *P* < 0.001, Fig. 2a, b). Further, the relationship between the VT–VO_2_ and the DLT–VO_2_ of the test cohort indicated that our DL algorithm was a clinically effective tool for estimating the threshold (*r* = 0.875, *P* < 0.001, Fig. 2e). The Bland–Altman plot revealed that the mean difference between the VT–VO_2_ and the DLT–VO_2_ in all three cohorts (derivation cohort: −0.14 mL/kg/min, validation cohort: −0.38 mL/kg/min, and test cohort: −0.05 mL/kg/min) was nonsignificant (*P* > 0.05, Fig. 2b, d, f). Therefore, these findings demonstrated that there was no bias between the mean values, which displayed strong agreements between the VT and the DLT.

### Subgroup analysis

The correlation coefficient between the VT–VO_2_ and the DLT–VO_2_ as the test cohort when stratified by patient characteristics was assessed (Supplementary Fig. 1). The correlation coefficients (*r*) were >0.7 in all the subgroups, and there were no major differences among stratified characteristics (*P* interaction > 0.05 for all).

## Discussion

In the present study, we found that the DL algorithm constructed with neural networks from single-lead ECG data during exercise enabled estimation of the VT in patients with CVDs. This study is unique in that we focused on the fact that electrical activity of the heart is dynamic during exercise and that the changes derived from hidden big data might resemble the feature of the VT.

DL is bringing a paradigm shift to healthcare, which is powered by increasing availability of healthcare data and rapid progress of analytic techniques. In recent years, the application of DL to cardiovascular medicine has advanced rapidly. Especially since the learning method called neural network has spread, we have made remarkable progress in precision. In the area of cardiovascular medicine, there are many applications of DL using neural networks; however, few reports are available in the area of cardiac rehabilitation. Myers et al.^16^ reported that deep neural networks could have application in the context of CPX for detecting cardiovascular outcome in patients with heart failure. Hearn et al.^17^ also described that harnessing neural networks with CPX data improved the prognostication of outcomes compared with the conventional prognostic method in patients with heart failure. These two studies implemented DL for the detection of prognosis. The strong point of our study is that our DL algorithm could recognize hidden features from single-lead ECG and estimate the VT, which was a clinically essential parameter for determining the level of exercise.

Our network structure combining one-dimensional convolution (1D-conv) and long–short term memory (LSTM) efficiently learned the complex time-series pattern of voltage in an ECG record and effectively estimated the time point of VT without the need for a respiratory gas analyzer. This combination of 1D-conv and LSTM has been shown to be effective in learning 12-lead ECG in our previous investigation^18^. The two-dimensional convolution network is useful in extracting information from a still image^19^. This is done by abstracting local information within the special axis of the images. The 1D-conv network does the similar abstraction but on only a single axis; in our case, it was a time axis. The LSTM is an improved version of the recurrent neural network, which is effective in learning time-series data. However, this network expands the steps of back propagation as the length of the input data increases. This causes the problem of vanishing gradient and makes the training computationally expensive. The combination of 1D-conv and LSTM may have been powerful for dealing with long time-series data by allowing abstraction of complex ECG patterns by 1D-conv and reducing the complexity of the data that the LSTM should learn. However, in order to deal with a larger number of voltages recorded in a single dataset, our study modified the network to form a more complex structure but was based on the same principal of the previously reported combination. We applied an ECG data length of 30 s to run our network structure combining 1D-conv and LSTM. We did not compare the model performance using other ECG length. A systematic research of the best ECG length may have further improved the model. Our study again shows that the combination of 1D-conv and LSTM is a powerful tool for dealing with time-series data of voltage recordings from ECG.

Cardiac rehabilitation defined by multidisciplinary professionals plays an important role in the disease management program for patients with CVDs, leading to improved exercise tolerance and quality of life, and reduced hospitalization^20^. Nevertheless, the application of cardiac rehabilitation for patients with CVDs is extremely low, especially in the outpatient setting^21–23^. Some factors contribute to such situations: complexity of CPX, time conflicts, and patients’ disinterest and uncertainty about the management of aerobic exercise in daily life^24^. Alternative methods are needed to facilitate the estimation of aerobic exercise thresholds and expand exercise therapy to the outpatient setting. We have previously demonstrated that a real-time evaluation of the HR variability (HRV) with single-lead ECG during CPX could be helpful for detecting the aerobic exercise threshold^25^. The study targeted patients who had myocardial infarction and sinus rhythm on ECG but not arrhythmia, such as AF, because the HRV analysis is not applicable in patients with irregular R–R intervals on ECG. In contrast, the method combined with the DL algorithm in the present study could be expanded to a wide range of CVDs patients, including those with AF during CPX. Further, if the algorithm of this study is mounted on wearable devices that can record ECGs, it can improve the persistence of cardiac rehabilitation programs in outpatients and relocate their bases from hospitals to other institutions, such as commercial fitness clubs or even patients’ homes.

Our findings should be interpreted in light of the following limitations. Firstly, the current analysis was performed in a single university hospital in Japan. The selection of patients who underwent CPX in the hospital may be biased. Further validation analyses using external datasets are necessary to establish the validity of our DL model. Second, variables of neural networks contained age, sex, and exercise time in addition to ECG data. Previous studies have suggested a correlation between VT, and age, sex, and peak VO_2_^26,27^. We also tested model excluding ECG data by training the same DL architecture with dummy ECG (all voltage for ECG was 0 for this analysis) to demonstrate the validity of the present study. In the model, there was also a correlation between DLT–VO_2_ and VT–VO_2_ of *r* = 0.771 (Supplementary Fig. 2). However, the correlation coefficient in the full model was significantly higher than that in the model without ECG data (the full model, *r* = 0.875 vs. the model without ECG data, *r* = 0.771, *P* < 0.05). These results suggest that ECG plays a crucial role in improving the accuracy of our model to achieve a good estimation for the VT. Third, the model requires the exercise time as an input. Therefore, it is not applicable to patients who cannot complete the CPX until exhaustion. Fourth, we could estimate the VT using DL including cardiac rhythm abnormalities (e.g., AF), and there was no significant deference in the DL model regardless of whether patients had AF (Supplementary Fig. 1). The number of patients with AF was limited (*n* = 25); therefore, further studies should be performed to assess the efficacy of our model in such patients. Finally, if used practically, the DL algorithm can estimate HR and work rate (Supplementary Fig. 3), but cannot calculate the values of VO_2_ or metabolic equivalents without a respiratory gas analyzer. Thus, it cannot replace the respiratory gas analyzer but may serve as a support system of CPX. Thus, our study is not yet an established method, and further proven experiences are needed to be used as a new estimating method in clinical practice.

In conclusion, this is the first study to show that the DL algorithm with neural networks using single-lead ECG data during CPX can estimate the VT in patients with CVDs. Given the difficult situation of estimating the VT, this method with DL could be helpful in estimating the VT.

## Methods

### Exercise testing protocol

The patients performed the test in the upright position on an electronically braked ergometer (Strength Ergo 8; Mitsubishi Electric Engineering Company, Tokyo, Japan). At first, the patients rested for 2 min on the ergometer until their heart rate (HR) and respiratory condition slowed down. Following a 2-min rest (rest phase), the patients performed a 2-min warm-up pedaling at 0 W (warm-up phase). The intensity was increased with a RAMP protocol ergometer (10–15 W/min), depending on the exercise capacities of each patient (exercise phase). The patients exercised with a progressive intensity until they could no longer maintain the pedaling rate (volitional exhaustion). After the exercise tests were terminated, the patients were instructed to stop pedaling and to stay on the ergometer for 3 min (recovery phase). The blood pressure was measured every minute with an indirect automatic manometer. Single-lead and 12-lead electrocardiograms were continuously recorded during whole test from the beginning of the rest phase to the end of the recovery phase.

### Respiratory gas analysis during CPX

The expired gas flows were measured using a breath-by-breath automated system (*V*_max_; Nihon Kohden, Tokyo, Japan). The respiratory gas exchange, including the ventilation, VO_2_, and carbon dioxide production, were monitored continuously and measured using a 30-s average. This system was subjected to a 3-way calibration process, involving a flow volume sensor, gas analyzer, and delay time calibration. VT was determined conventionally using the procedures described by Gaskill et al. (i.e., the ventilatory equivalent, excess carbon dioxide, and modified V-slope methods)^28^. The peak VO_2_ was calculated as the average oxygen consumption during the last 30 s of exercise. The ventilation/carbon dioxide (ventilator efficiency) slope (VE–VCO_2_ slope) was based on data from the onset of exercise to the respiratory compensation point, and it was obtained by performing a linear regression analysis of the data acquired throughout the entire period of exercise^29,30^. The respiration quotients at VT and peak exercise were measured.

### Electrocardiographic sampling, preprocessing data, and construction of the DL model

**Fig. 3:**
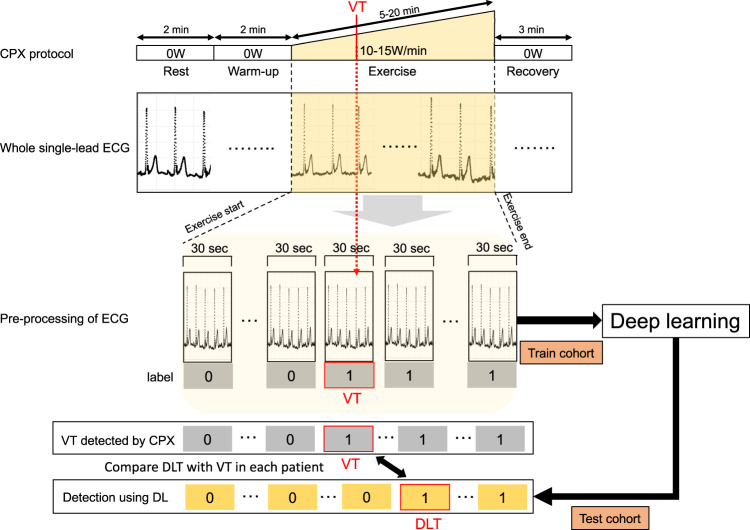
Data conversion and deep learning for the estimation of VT from single-lead electrocardiography data. Schematic illustration of the pre-processing of electrocardiography and application of deep learning. ECG electrocardiography, VT ventilatory threshold, DLT deep learning threshold, DL deep learning, VO_2_ oxygen uptake, CPX cardiopulmonary exercise testing.

**Fig. 4:**
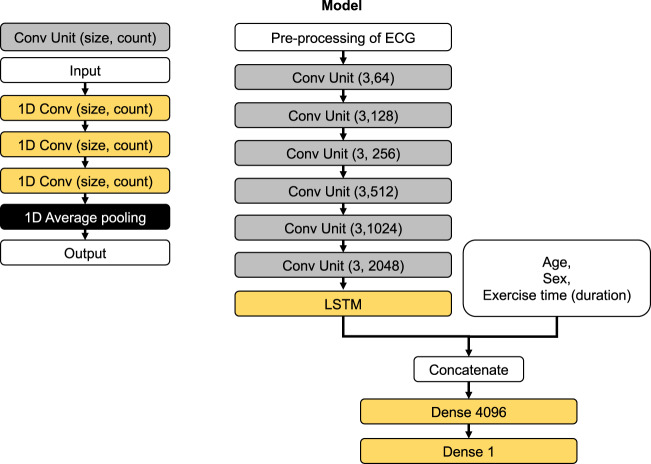
Structure of the neural network in our deep learning model. Schematic illustration of the neural network model. The details of each cell in the network are shown in the left panel, and the overall network structure is shown in the right panel. Con convolution, ECG electrocardiography, 1D Conv one-dimensional convolution, LSTM long short-term memory, 1D max pooling one-dimensional max pooling.

**Fig. 5:**
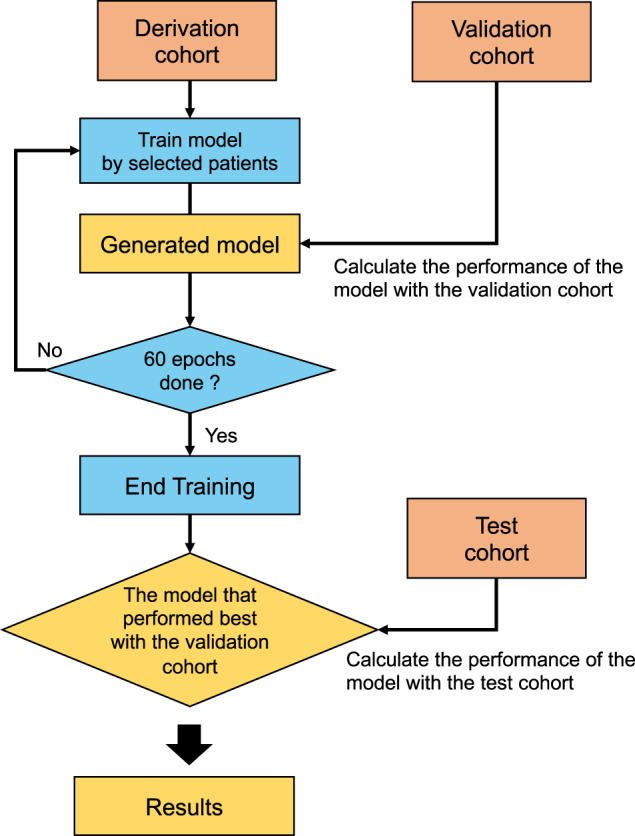
Training and testing of the model. Schematic illustration of the process of training and testing the model. The model was trained with data from the derivation cohort, and the performance of each model was calculated using data from the validation dataset on the end of each epoch. The final model was chosen as the model that performed best for the 60 epochs in the validation cohort. The performance of the final model was calculated only once using data from the testing dataset.

Among 260 cardiovascular patients, 97 (37.3%), 72 (27.7%), and 91 (35.0%) patients were randomly assigned to derivation, validation, and test cohorts, respectively. A combination of the derivation and validation cohorts were grouped with the train cohort (Fig. 1). The overall process of this study is illustrated in Fig. 3. The single-lead ECG data were stored as measurements of time-series voltage with a sampling rate of 1000 Hz by the LRR-03 (Crosswell, Yokohama, Japan). The conversion of ECG data to matrices was done using a previously published method with slight modification^18^. The ECG data from the beginning to the end of exercise phase (the ECG data painted yellow in Fig. 3) were extracted, and divided into multiple sections of 30 s. Each section was labeled independently as before VT (0) and including or after VT (1). Each labeled section of the 30 s-series of ECG data along with the patients’ demographic data (age and sex) and exercise time (duration) was independently fed into the network for training. The network structure of the DL model is shown in Fig. 4. We constructed the structure with a combination of one-dimensional (1D) convolution and LSTM to deal with the time-series data points in single-lead ECG data, converted to a one-dimensional matrix containing the recorded voltage for each 1 ms. The neural network was constructed and trained using the Keras framework (https://keras.io) with TensorFlow^31^ as backend. The neural network was trained using the back-propagation supervised training algorithm. The loss function of binary cross entropy was minimized using the RMSprop optimizer (https://www.coursera.org/learn/neural-networks/home/welcome). The network was trained for 60 epochs, and the model that performed best with the validation cohort was selected as the final model (Fig. 5). The performance of the final model was tested only once on the test dataset to confirm that the model was not over-fitted.

### DL threshold

The DLT was defined as an initial time zone of 30 s-series including the VT estimated by the DL model. We validated the relationships between VT–VO_2_ and DLT–VO_2_ as the derivation, validation, and test cohorts to confirm that the DLT was the good threshold for clinically estimating the VT (Fig. 3).

### Statistical analyses

The results are represented as the mean ± standard deviation for continuous variables and as a percentage for categorical variables, as appropriate. The relationships between the studied methods of the VT and the DLT as derivation, validation, and test cohorts were investigated by using the Pearson correlation coefficient test. The Bland–Altman technique was applied to verify the similarities between the different methods (VT and DLT)^32^. This comparison was a graphical representation of the difference between the methods and the average of these methods. In addition, we stratified patients into groups based on disease history (chronic heart failure, coronary artery disease, and pulmonary hypertension), left ventricular ejection fraction, rhythm of AF during CPX, and prescription of a β blocker, and estimated the relationship between the subgroups in the test cohort. Correlation coefficients between the full model and the model without ECG were compared using the correlation coefficient difference test. All probability values were two-tailed, and *P* values < 0.05 were considered to be statistically significant. All statistical analyses were performed with SPSS version 25.0 software (IBM Corp., Armonk, NY).

### Ethics and registration

The study protocol was approved by the Institutional Review Board of Keio University School of Medicine (permission number: 2014023) and conducted in accordance with the Declaration of Helsinki. All patients provided written informed consent.

### Reporting summary

Further information on research design is available in the Nature Research Reporting Summary linked to this article.

## Supplementary information

Supplementary Information

Reporting Summary

## Data Availability

The data that support the findings of this study are available from the corresponding author upon reasonable request.

## Code availability

The codes that support the findings of this study are available from the corresponding author upon reasonable request.

## References

[1] Schnohr P, O’Keefe JH, Marott JL, Lange P, Jensen GB (2015). Dose of jogging and long-term mortality: the Copenhagen City Heart Study. J. Am. Coll. Cardiol..

[2] Wen CP (2011). Minimum amount of physical activity for reduced mortality and extended life expectancy: a prospective cohort study. Lancet.

[3] Warburton DE, Nicol CW, Bredin SS (2006). Prescribing exercise as preventive therapy. CMAJ.

[4] Warburton DE, Nicol CW, Bredin SS (2006). Health benefits of physical activity: the evidence. CMAJ.

[5] Strike PC (2006). Triggering of acute coronary syndromes by physical exertion and anger: clinical and sociodemographic characteristics. Heart.

[6] Shiraishi Y (2018). Impact of triggering events on outcomes of acute heart failure. Am. J. Med..

[7] Piepoli MF (2016). 2016 European Guidelines on cardiovascular disease prevention in clinical practice: The Sixth Joint Task Force of the European Society of Cardiology and Other Societies on Cardiovascular Disease Prevention in Clinical Practice (constituted by representatives of 10 societies and by invited experts)Developed with the special contribution of the European Association for Cardiovascular Prevention & Rehabilitation (EACPR). Eur. Heart J..

[8] Haskell WL (2007). Physical activity and public health: updated recommendation for adults from the American College of Sports Medicine and the American Heart Association. Circulation.

[9] Krittanawong C, Zhang H, Wang Z, Aydar M, Kitai T (2017). Artificial intelligence in precision cardiovascular medicine. J. Am. Coll. Cardiol..

[10] Attia ZI (2019). An artificial intelligence-enabled ECG algorithm for the identification of patients with atrial fibrillation during sinus rhythm: a retrospective analysis of outcome prediction. Lancet.

[11] Zhang J (2018). Fully automated echocardiogram interpretation in clinical practice. Circulation.

[12] Hong, Y. et al. Deep learning-based stenosis quantification from coronary CT angiography. In *Medical Imaging 2019: Image Processing* (2019). 10.1117/12.2512168 PMC6874408 31762536

[13] Betancur J (2018). Deep learning for prediction of obstructive disease from fast myocardial perfusion SPECT: a multicenter study. JACC Cardiovasc. Imaging.

[14] Bai W (2018). Automated cardiovascular magnetic resonance image analysis with fully convolutional networks. J. Cardiovasc. Magn. Reson..

[15] Sengupta PP, Kulkarni H, Narula J (2018). Prediction of abnormal myocardial relaxation from signal processed surface ECG. J. Am. Coll. Cardiol..

[16] Myers J (2014). A neural network approach to predicting outcomes in heart failure using cardiopulmonary exercise testing. Int J. Cardiol..

[17] Hearn J (2018). Neural networks for prognostication of patients with heart failure. Circ. Heart Fail..

[18] Goto S (2019). Artificial intelligence to predict needs for urgent revascularization from 12-leads electrocardiography in emergency patients. PLoS ONE.

[19] Esteva A (2017). Dermatologist-level classification of skin cancer with deep neural networks. Nature.

[20] Davidson PM (2010). Can a heart failure-specific cardiac rehabilitation program decrease hospitalizations and improve outcomes in high-risk patients?. Eur. J. Cardiovasc. Prev. Rehabil..

[21] Turk-Adawi K, Sarrafzadegan N, Grace SL (2014). Global availability of cardiac rehabilitation. Nat. Rev. Cardiol..

[22] Park LG, Schopfer DW, Zhang N, Shen H, Whooley MA (2017). Participation in cardiac rehabilitation among patients with heart failure. J. Card. Fail..

[23] Kamiya K (2019). Nationwide survey of multidisciplinary care and cardiac rehabilitation for patients with heart failure in Japan—an analysis of the AMED-CHF study. Circ. J..

[24] Grace SL (2009). Barriers to cardiac rehabilitation: does age make a difference?. J. Cardiopulm. Rehabil. Prev..

[25] Shiraishi Y (2018). Real-time analysis of the heart rate variability during incremental exercise for the detection of the ventilatory threshold. J. Am. Heart Assoc..

[26] Haruki I, Akira K, Taniguchi K, Marumo F (1989). Severity and pathophysiology of heart failure on the basis of anaerobic threshold (AT) and related parameters. Jpn. Circ. J..

[27] Arena R, Sietsema KE (2011). Cardiopulmonary exercise testing in the clinical evaluation of patients with heart and lung disease. Circulation.

[28] Gaskill S (2001). Validity and reliability of combining three methods to determine ventilatory threshold. Med. Sci. Sports Exerc..

[29] Francis D (2000). Cardiopulmonary exercise testing for prognosis in chronic heart failure: continuous and independent prognostic value from VE/VCO2 slope and peak VO2. Eur. Heart J..

[30] Clark AL, Poole-Wilson PA, Coats AJS (1992). Relation between ventilation and carbon dioxide production in patients with chronic heart failure. J. Am. Coll. Cardiol..

[31] Martín Abadi, A. A. et al. TensorFlow: Large-scale machine learning on heterogeneous systems. Software available from tensorflow.org (2015).

[32] Bland J, Altman D (1986). Statistical methods for assessing agreement between two methods of clinical measurement. Lancet.

