# Supplementary material for: Feasibility of the deep learning method for estimating the ventilatory threshold with electrocardiography data
Source: NPJ Digit Med. 2020 Oct 29;3:141. doi: 10.1038/s41746-020-00348-6 (PMC7596490; doi:10.1038/s41746-020-00348-6)
Supplement: Supplementary file 1 — Supplementary Information [file 41746_2020_348_MOESM1_ESM.pdf]

SUPPLEMENTARY INFORMATION

Supplementary Table 1. Detailed classification of medical history

	All patients (n = 260)	Train cohort (n = 169)	Test cohort (n = 91)
Chronic heart failure	121 (46.5%)	78 (46.2%)	43 (47.3%)
LVEF ≥50%	44 (16.9%)	27 (16%)	17 (18.7%)
LVEF <50%	77 (29.6%)	51 (30.2%)	26 (28.6%)
Coronary artery disease	100 (38.4%)	70 (41.4%)	30 (33.0%)
Post-PCI	95 (36.5%)	66 (39.1%)	29 (31.9%)
OMT	5 (1.9%)	4 (2.4%)	1 (1.1%)
Pulmonary hypertension	41 (15.8%)	22 (13.0%)	19 (20.9%)
CTEPH	38 (14.6%)	20 (11.8%)	18 (19.8%)
IPAH	3 (1.2%)	2 (1.2%)	1 (1.1%)
Arrhythmias	37 (14.2%)	14 (15.4%)	23 (13.6%)

Paroxysmal AF	4 (1.5%)	1 (0.6%)	3 (3.3%)
Persistent AF	23 (8.8%)	15 (8.9%)	8 (8.8%)
Post AF ablation	4 (1.5%)	1 (0.6%)	3 (3.3%)
The others	6 (2.3%)	6 (3.6%)	0

LVEF, left ventricular ejection fraction; PCI, percutaneous coronary intervention; OMT, optimal medical therapy; CTEPH, chronic thromboembolic pulmonary hypertension; IPAH, idiopathic pulmonary hypertension; AF, atrial fibrillation.

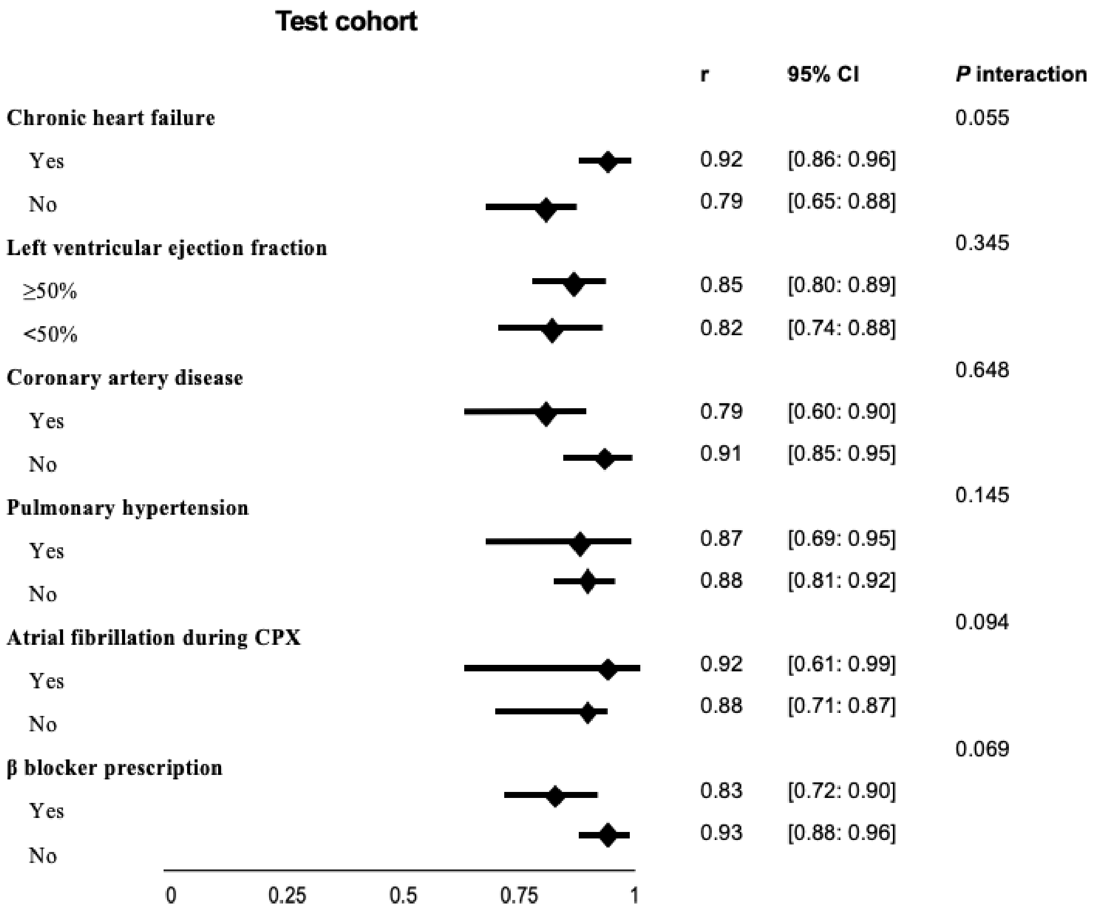

Supplementary Figure 1: The correlation coefficient in stratified subgroups

Forest plot show that the correlation coefficient between VT-VO_2 and DLT-VO_2 as test cohort compared with that is stratified with patients' characteristics, atrial fibrillation during CPX, and the prescription of β blocker.

CPX, cardiopulmonary exercise testing; CI, confidence interval; VT, ventilatory threshold; VO_2, oxygen uptake; AIT, artificial intelligence threshold.

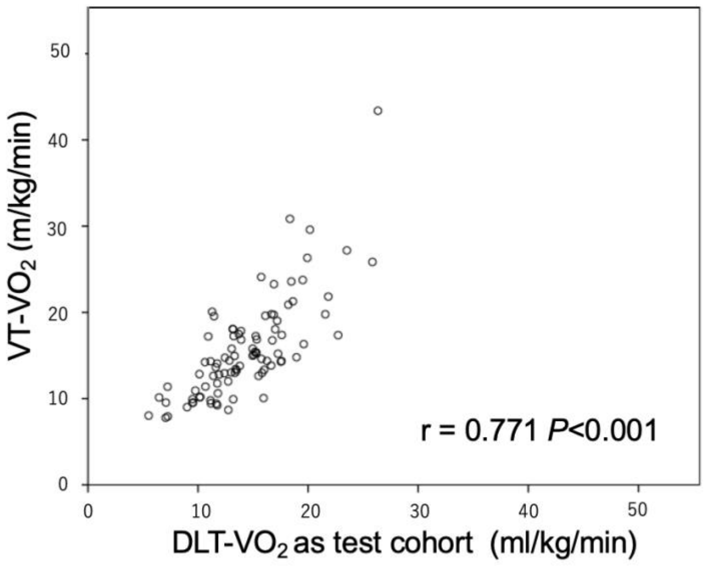

Supplementary Figure 2: Relationship between the VT-VO_2 and the DLT-VO_2 for the test cohort in the model without ECG data.

DLT, deep learning threshold; ECG, electrocardiography; VT, ventilatory threshold; VO_2, oxygen uptake.

(a)

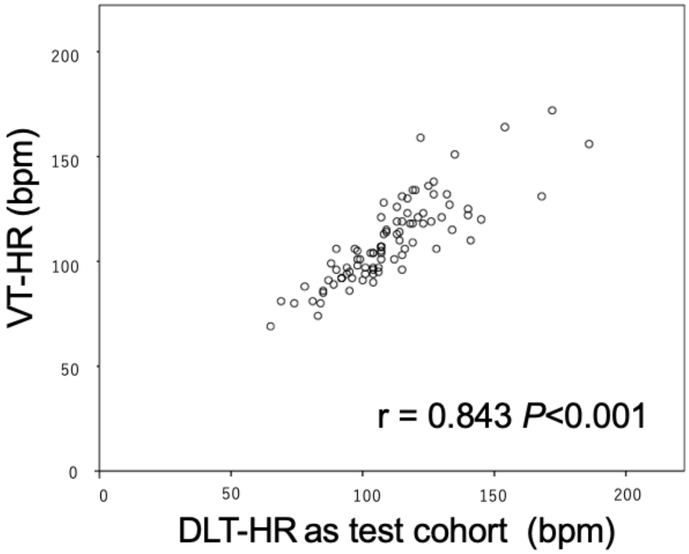

(b)

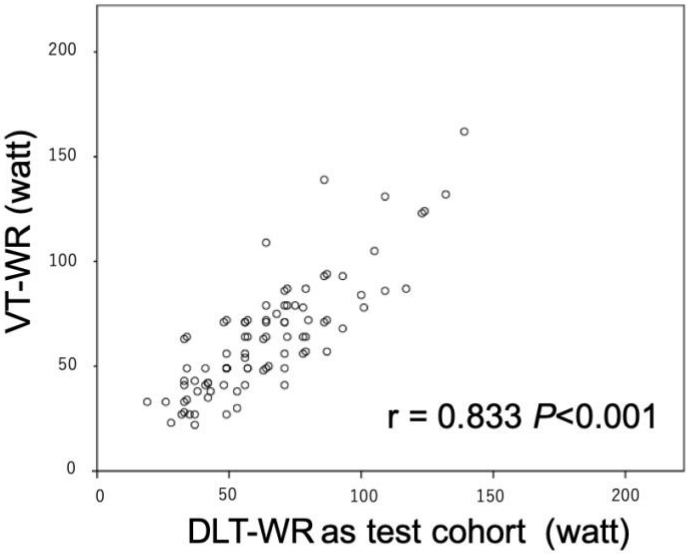

Supplementary Figure 3: Relationship between the heart rates and the work rates at the VT and DLT for the test cohort.

(a) Relationship between the heart rates at the VT and DLT.

(b) Relationship between the work rates at the VT and DLT.

DLT, deep learning threshold; HR, heart rate; VT, ventilatory threshold; WR, work rate
