# Supplementary material for: Feasibility of the deep learning method for estimating the ventilatory threshold with electrocardiography data
Source: NPJ Digit Med. 2020 Oct 29;3:141. doi: 10.1038/s41746-020-00348-6 (PMC7596490; doi:10.1038/s41746-020-00348-6)
Supplement: Supplementary file 2 — Reporting Summary [file 41746_2020_348_MOESM2_ESM.pdf]

nature research

Corresponding author(s): Yoshinori Katsumata

Last updated by author(s): Sep 29, 2020

Reporting Summary

Nature Research wishes to improve the reproducibility of the work that we publish. This form provides structure for consistency and transparency in reporting. For further information on Nature Research policies, see our Editorial Policies and the Editorial Policy Checklist.

Statistics

For all statistical analyses, confirm that the following items are present in the figure legend, table legend, main text, or Methods section.

n/a	Confirmed	
☐	☒	The exact sample size (*n*) for each experimental group/condition, given as a discrete number and unit of measurement
☒	☐	A statement on whether measurements were taken from distinct samples or whether the same sample was measured repeatedly
☐	☒	The statistical test(s) used AND whether they are one- or two-sided<br>*Only common tests should be described solely by name; describe more complex techniques in the Methods section.*
☒	☐	A description of all covariates tested
☒	☐	A description of any assumptions or corrections, such as tests of normality and adjustment for multiple comparisons
☐	☒	A full description of the statistical parameters including central tendency (e.g. means) or other basic estimates (e.g. regression coefficient) AND variation (e.g. standard deviation) or associated estimates of uncertainty (e.g. confidence intervals)
☒	☐	For null hypothesis testing, the test statistic (e.g. *F*, *t*, *r*) with confidence intervals, effect sizes, degrees of freedom and *P* value noted<br>*Give P values as exact values whenever suitable.*
☒	☐	For Bayesian analysis, information on the choice of priors and Markov chain Monte Carlo settings
☒	☐	For hierarchical and complex designs, identification of the appropriate level for tests and full reporting of outcomes
☒	☐	Estimates of effect sizes (e.g. Cohen's *d*, Pearson's *r*), indicating how they were calculated

Our web collection on statistics for biologists contains articles on many of the points above.

Software and code

Policy information about availability of computer code

Data collection	We do not use custom software for data collection.
Data analysis	All the models were trained with Keras 2.3.0 on a Tensorflow 1.14.0 backend on Python 3.7.3.

For manuscripts utilizing custom algorithms or software that are central to the research but not yet described in published literature, software must be made available to editors and reviewers. We strongly encourage code deposition in a community repository (e.g. GitHub). See the Nature Research guidelines for submitting code & software for further information.

Data

Policy information about availability of data

All manuscripts must include a data availability statement. This statement should provide the following information, where applicable:

- Accession codes, unique identifiers, or web links for publicly available datasets
- A list of figures that have associated raw data
- A description of any restrictions on data availability

The data that support the findings of this study are available from the corresponding author upon reasonable request.

Field-specific reporting

Please select the one below that is the best fit for your research. If you are not sure, read the appropriate sections before making your selection.

☒ Life sciences ☐ Behavioural & social sciences ☐ Ecological, evolutionary & environmental sciences

For a reference copy of the document with all sections, see nature.com/documents/nr-reporting-summary-flat.pdf

Life sciences study design

All studies must disclose on these points even when the disclosure is negative.

Sample size	The sample-size calculation was not performed in this study. The number of samples was determined according to the patients' selection protocol from the data of the available time period.
Data exclusions	The three exclusion criteria are as follows: patients with missing data, patients terminated by physician’s decision before patients reached their peak exercise, and patients who had pacing rhythm for pacemaker implantation. The second exclusion criteria was established to exclude data in which the exercise load was arbitrarily terminated. Moreover, since this study analyzed the electrical signals of living organisms, we excluded ECG data in which mechanical electrical signals were input by the pacing device.
Replication	We tested the replication of the result in 2 patient population, which was separated randomly (validation and test dataset).
Randomization	In this study, all patients were randomly divided into three groups: "derivation cohort", "validation cohort", and "test cohort". The randomization was done with the random number generator of numpy version 1.9.0.
Blinding	This study is a retrospective study, and the disclosure of the blinding of the allocation is not relevant.

Reporting for specific materials, systems and methods

We require information from authors about some types of materials, experimental systems and methods used in many studies. Here, indicate whether each material, system or method listed is relevant to your study. If you are not sure if a list item applies to your research, read the appropriate section before selecting a response.

Materials & experimental systems

n/a	Involved in the study	
☒	☐	Antibodies
☒	☐	Eukaryotic cell lines
☒	☐	Palaeontology and archaeology
☒	☐	Animals and other organisms
☐	☒	Human research participants
☒	☐	Clinical data
☒	☐	Dual use research of concern

Methods

n/a	Involved in the study	
☒	☐	ChIP-seq
☒	☐	Flow cytometry
☒	☐	MRI-based neuroimaging

Human research participants

Policy information about studies involving human research participants

Population characteristics	The patients were all Japaneses, predominantly men (73.1%), with an average age of 58.9±14.6 years. They all have cardiovascular diseases (chronic heart failure, coronary artery disease, pulmonary hypertension, and/or arrhythmias).
Recruitment	This study was conducted in a single-center university hospital and is considered to contain patient selection bias. This is an important limitation and is clearly stated in the manuscript.
Ethics oversight	The Institutional Review Board of Keio University School of Medicine

Note that full information on the approval of the study protocol must also be provided in the manuscript.
